# Tic Doloureux

**Published:** 1853-10

**Authors:** 


*Tic Doloureux.*—Mr. Beaumont recommends the following combination in this distressing affection:

℞ Ferri potassio-tartratis, ℈ij.
Vini opii, gtt. lxxx.
Aquæ cinnamomi, ℥viij.
M.

Fiat mistura. Three tablespoonfuls thrice daily.—*Association Med. Jour.*

